# The Utility of Fecal Calprotectin in the Real-World Clinical Care of Patients with Inflammatory Bowel Disease

**DOI:** 10.1155/2016/2483261

**Published:** 2016-09-28

**Authors:** Esmail Abej, Wael El-Matary, Harminder Singh, Charles N. Bernstein

**Affiliations:** ^1^Section of Gastroenterology, Department of Internal Medicine, Winnipeg, MB, Canada; ^2^University of Manitoba and the University of Manitoba IBD Clinical and Research Centre, Winnipeg, MB, Canada; ^3^Pediatric Gastroenterology, Department of Pediatrics and Child Health, Winnipeg, MB, Canada

## Abstract

*Objectives.* To determine the relationship between fecal calprotectin (FCAL) and imaging studies and other biochemical inflammatory markers and the impact of FCAL measurements on decision-making in IBD patient management in usual clinical practice.* Methods.* 240 persons with IBD were enrolled. The correlation between FCAL values and other markers for disease activity such as serum albumin (alb), hemoglobin (Hg), and C-reactive protein (CRP) and diagnostic imaging or colonoscopy was examined. FCAL ≥ 250 mcg/g of stool was considered a positive result indicating active IBD.* Results.* 183 stool samples (76.3%) were returned. The return rate in the pediatric and adult cohorts was 91% (*n* = 82) and 67.3% (*n* = 101), respectively (*P* < 0.0001). Positive FCAL was associated with colonoscopy findings of active IBD (*P* < 0.05), low albumin (*P* < 0.05), anemia (*P* < 0.01), and elevated CRP (*P* < 0.01). There was no significant difference for FCAL results by outcomes on small bowel evaluation among the 21 persons with small bowel CD. Most persons (87.5%) with normal FCAL and no change in therapy remained in remission during subsequent 3 months.* Conclusions.* FCAL is a useful marker of disease activity and a valuable tool in managing persons with IBD in clinical practice. Clinicians have to be cautious in interpreting FCAL results in small bowel CD.

## 1. Introduction

Inflammatory bowel disease (IBD) is a group of closely related diseases of immune dysregulation including ulcerative colitis (UC), Crohn's disease (CD), and IBD type unclassified (IBD-U). IBD is a chronic disease with remissions and relapses that requires long-term monitoring and runs a variable course. Poor control of ongoing inflammation has been associated with poor outcomes [1]. In clinical practice, disease activity in IBD has been monitored using several markers with variable levels of accuracy and invasiveness. Biological biomarkers such as C-reactive protein (CRP) and serum albumin have low sensitivity and specificity and gastroenterologists do not rely on them exclusively in monitoring the course of IBD or in diagnosing a flare [2]. Diagnostic imaging and colonoscopy have been the gold standards for defining the state of disease activity. If treatment to target of mucosal healing [3] in IBD is to be widely adopted, monitoring inflammation necessitates a greater use of these invasive and/or expensive tools, unless less invasive tools are found.

Calprotectin is a calcium and zinc binding protein found in the cytosol of human neutrophils and macrophages. It is released extracellularly in times of cell stress or damage and can be detected within feces and thus can be used as a sensitive marker of intestinal inflammation. Fecal calprotectin (FCAL) has emerged as a potential noninvasive, inexpensive, and more accurate tool than serological measures in monitoring IBD disease activity and diagnosing a flare. It has also been found to be useful in differentiating irritable bowel syndrome (IBS) from IBD [4, 5]. Although FCAL has been available for some time and has been shown to correlate well with endoscopy [6–8] the applications of FCAL in monitoring inflammation in IBD in clinical practice have not been standardized and the impact of addition of FCAL on the diagnostic and monitoring armamentarium in IBD is not well defined. The aim of our study was to determine the relationship between FCAL measurements and other traditional diagnostic tests in IBD: both imaging and serological markers and the impact of FCAL results on the decision-making in management of persons with IBD in “real-world” clinical practice.

## 2. Methods

### 2.1. Patients

In this single-centre cross-sectional study, 240 persons with IBD seen in the Gastroenterology Outpatient Clinics of three university hospital-based gastroenterologists (two adult and one pediatric) for either routine follow-up or new symptoms, between August 2013 and February 2016, were asked to bring in a stool sample for FCAL measurements. The samples were collected at home and processed at the laboratory of the University of Manitoba IBD Clinical and Research Centre. Persons with IBD aged four or above and with confirmed diagnoses of IBD by endoscopy and/or radiography were included. FCAL was done using the Quantum Blue® Lateral Flow Reader and within 24 hours of stool collection. FCAL ≥ 250 mcg/g was considered a positive test indicative of active IBD.

Participating clinicians were given a questionnaire to collect patients' demographics, diagnoses, physician global assessment (PGA), and the impact of FCAL testing on the management of patients in terms of plans for investigation(s) or changes in therapy. The clinicians determined at the time of ordering FCAL whether they would undertake other investigations, but they also could determine to pursue other investigations once the FCAL result became available. Once the FCAL result was available the clinicians recorded whether and how it changed their management. The ordering of diagnostic imaging and blood work was left to the discretion of the clinicians. Clinicians had the FCAL test results within one week.

### 2.2. Data Collection

Medical records of all included persons were reviewed and data were collected on serum levels of C-reactive protein (CRP), albumin, and haemoglobin (Hg) done within two weeks of collecting stool samples. In our local laboratory an abnormal CRP was equal to or greater than 8 mg/L. A low level of serum albumin was equal to or less than 33 g/L. We used World Health Organization (WHO) definition of anemia, which included the following values: male age ≥ 15 yr Hg < 140 g/L, female age ≥ 15 yr Hg < 120 g/L, children 12–14 yr < 120 g/L, and children 4–11 yr Hb < 115 g/L. Abdominal diagnostic imaging, colonoscopies, and flexible sigmoidoscopies were performed within one month of collecting stool samples. This time frame was chosen because MRE, CTE, or endoscopy can be generally arranged within one month in our centre. We reviewed reports of all abdominal diagnostic imaging (computed tomographic enterography (CTE) and magnetic resonance enterography (MRE)) and used the radiologic report from the gastrointestinal radiologist to determine disease activity. The definition of active disease on CTE included findings such as stricturing with mucosal hyperenhancement; bowel wall thickening; or thumb printing and findings on MRE included abnormal enhancement, wall thickening, or ulcerations. The presence of erythema, loss of vascularity, friability, or ulcerations were included in the definition of active disease on endoscopy.

In order to determine the value of normal FCAL in real-world practice, patients who had normal FCAL measurements and no investigation or change in therapy were followed up prospectively for 3 months to assess relapse rate which was defined by hospitalization, change in therapy, or endoscopy or imaging showing active disease.

### 2.3. Statistical Analysis

Nominal variable comparisons were performed using Fisher's Exact test. A *P* value <0.05 was considered significant. Odds ratios (ORs) and 95% confidence interval (CI) of the multiple variables of interest in relation to FCAL measurement results were calculated. Kruskal-Wallis test was used to compare medians of ages of returners of stool samples versus nonreturners.

### 2.4. Ethics

The study protocol was approved by the University of Manitoba Health Research Ethics Committee.

## 3. Results

A total of 183 persons returned stool samples (of 240 who were asked to bring in a stool sample; adults *n* = 150 and children *n* = 90) (Table 1). The overall return rate was 76.3%. The return rate was higher in the pediatric cohort (*n* = 82, 91%) compared to the adult cohort (*n* = 101, 67%, *P* < 0.0001). The adult subjects (age ≥ 18) who did not return stool samples were younger than those who returned the stool samples (median age of 38 years versus 47, *P* = 0.017); however a similar proportion of sample returnees and non returnees were female (63.2% versus 64.4%) (Table 2). Ninety-nine subjects had a positive FCAL (≥250 mcg/g).

### 3.1. Imaging

Twenty-three subjects underwent CTE or MRE. Of 9 persons with active disease on CTE or MRI, only 6 had a positive FCAL. Of 11 subjects with a positive FCAL who underwent imaging, only 6 had active disease on imaging. A positive FCAL was not significantly associated with radiologic evidence of active disease (*P* = 0.31).

### 3.2. Lower Gastrointestinal Endoscopy

Clinicians chose to undertake colonoscopy or flexible sigmoidoscopy in 33, either based on their assessment or because of elevated FCAL. Twenty-one (67%) with evidence of active disease on endoscopy had positive FCAL (*P* = 0.01, odds ratio (OR), 8.79; 95% CI 1.54–65.55). Hence positive FCAL had 80.9% sensitivity and 69.2% specificity in comparison to the findings of active disease on endoscopy.

### 3.3. Serological Biomarkers

150 subjects had serum albumin measured. Seventeen out of 20 who had low serum albumin also had positive FCAL (*P* = 0.015, OR 4.66; 95% CI, 1.31–5.96). 147 subjects had serum Hg measured. Of 56 persons with anemia 40 had positive FCAL (*P* = 0.006, OR 2.74; 95% CI 1.31–5.96). 132 had serum CRP measured. Of 36 with high CRP, 28 had positive FCAL (*P* = 0.006, OR 3.46; 95% CI 1.36–9.73). Of persons with positive FCAL (*n* = 99), 78 were clinically diagnosed to have active disease including mild (*n* = 52), moderate (*n* = 23), and severely active disease (*n* = 3) as assessed by the participating clinicians using the PGA scale before the FCAL testing was performed (*P* = 0.0004, OR 3.68; 95% CI 1.86–7.48) (Table 3). The mean FCAL level for mild disease was 1078 ± 616 and 1212 ± 565 for moderate disease. These were not significantly different.

### 3.4. Small Bowel CD

Of the twenty-one persons with small bowel CD and either diagnostic imaging (*n* = 10) or endoscopy (*n* = 11, 3 of which had video capsule endoscopy), there was no significant difference in outcome by FCAL results (*P* = 0.73 for imaging and *P* = 0.39 for endoscopy).

For the 99 persons with a positive FCAL test clinicians made a change in therapy or investigations in (*n* = 86) 87% (Table 5). On the other hand, based on a negative FCAL (*n* = 84), clinicians made no change in therapy or further investigations in (*n* = 74) 88% (Table 4).

Of the 74 persons who had normal FCAL (<250 mcg/g) and had no change in therapy or investigations, 8 (12.5%) had a flare within 3 months of the date FCAL test. Three of them required hospitalization. Five of these 8 persons had FCAL test results between 150 and 249 mcg/g. Six additional persons had FCAL results between 150 and 249 mcg/g and they did not have a flare of symptoms within 3 months. Hence, a total of eleven persons had a FCAL result of 150–249 mcg/g and five of eleven had a flare within 3 months.

## 4. Discussion

FCAL is a surrogate marker of intestinal mucosal inflammation. Several studies and meta-analyses have shown that FCAL is useful for discriminating IBD from other diseases [9, 10] and predicting relapse of patients with IBD in remission [11]. Assessment of disease activity requires timely endoscopy or diagnostic imaging and both of these methods have their limitations in cost, invasiveness, or availability in a timely fashion. Little is known about the adherence of IBD patients to FCAL testing especially in the adult populations. Our study showed excellent adherence rate in the paediatric cohort (91%) and an incomplete rate in adults (67%). We found that there was a strong correlation between FCAL and serum markers and also endoscopy but a poor correlation with cross-sectional imaging, although the sample size for cross-sectional imaging was small. We also found that clinicians put a lot of faith in the FCAL results and many with negative FCAL results did not get further investigations. It is difficult to discern what role the FCAL result had in the clinicians not pursuing investigations or therapy changes as opposed to their management consideration being based on a composite of other testing, as well.

Only 12.5% of persons who had no change in therapy or investigations based on negative FCAL results had a flare within 3 months. Allowing for the lack of controls and short follow-up time, this may indicate that the FCAL test can be used reliably in clinical practice. Given the noninvasive nature, quick turnaround of FCAL test, and good correlation with endoscopy in colonic inflammatory bowel diseases as we have shown in this study, FCAL can help in monitoring disease activity and may be used as a tool along with other serologic and clinical measures to guide further investigation and therapy. While adherence rates in children were excellent they could be improved in adults.

We used a cut-off of less than 250 mcg/g as normal for an IBD population. In a meta-analysis including 13 studies (744 patients with UC and 727 with CD) using a cut-off value of 250 mcg/g the areas under the curve values for diagnostic accuracy of disease activity were 0.89 (95% CI, 0.86–0.92), 0.93 (0.89–0.97), and 0.88 (0.83–0.93) in IBD, UC, and CD groups, respectively. The pooled sensitivity was 0.80 (0.76–0.84) and specificity was 0.82 (0.77–0.86). It was concluded that FCAL was a reliable marker for assessing IBD disease activity and may have greater ability to evaluate disease activity in UC than CD [12]. While we adopted the cut-off value of 250 mcg/g as abnormal, considering that five of eleven persons with a FCAL of 150–249 mcg/g experienced a flare within 3 months and these were the majority of persons who flared in the normal FCAL group, we may need to reconsider the optimal cut-off for normal in IBD. More research is needed to determine if 150 or 250 mcg/g should be the cut-off in IBD.

Data on assessing disease activity in CD comparing FCAL with MRE or CRE results are conflicting. Quaia et al. studied 91 patients with CD who underwent MRE. They compared MRE findings to the Crohn's Disease Endoscopic Index of Severity (CDEIS) and with histologic analysis of those who underwent elective small bowel resection (*n* = 30). The bowel wall T2 hyperintensity (odds ratio [OR], 9.20; 95% CI, 2.71–31.19) and total length of disease (OR, 1.29; 95% CI, 1.11–1.49) were found as the best independent predictors of active CD. CDAI, C-reactive protein, and FCAL were not found to be independent predictors of active CD [13]. Cerrillo et al. examined the relation between FCAL level and disease activity on MRE in 120 patients with ileal CD. The Magnetic Resonance Index of Activity score was significantly associated with FCAL levels (*P* = 0.01), with a modest overall correlation (Spearman's *r* = 0.56, *P* = 0.001). FCAL reflected MRE inflammatory activity with an area under the receiver operating characteristic curve of 0.91 (CI, 0.85–0.96; *P* = 0.001). A cut-off value of 166.5 mg/g had 90% sensitivity, 74% specificity, 89% positive predictive value, and 76% negative predictive value for diagnosis of inflammation [14]. Elsewhere a MRE global score (MEGS) and a CD activity score (CDAS) were modestly but significantly correlated with FCAL (*r* = 0.46, *P* < 0.001) and (*r* = 0.39, *P* = 0.001), respectively [15]. Allowing for the small number of patients (*n* = 21), our study did not show a signification association between FCAL levels and disease activity on MRE/CTE. To our knowledge no systematic review or meta-analysis has addressed the question of small bowel CD activity and FCAL levels. Clinicians have to be cautious in interpreting FCAL results in this subtype of CD patients.

The limitations to our study were that the same testing was not undertaken in all subjects to fully determine the correlations between the FCAL and various outcomes, especially cross-sectional imaging. However, at study outset, we were particularly interested in determining how clinicians would use FCAL results. A positive FCAL often triggered a clinical response, either more investigations or a change in therapy. Unfortunately, not having done imaging in all subjects, we cannot discern how many false negative FCAL tests are represented within our sample. Nonetheless, our results do show how much faith clinicians have placed in FCAL based on the medical literature since all participating clinicians were utilizing FCAL for the first time in their practices.

## 5. Conclusions

Our study represents a snapshot of how FCAL testing is used in monitoring disease activity in IBD in clinical practice outside the context of clinical trials. We found that in a referral population of persons with IBD, positive FCAL was significantly associated with abnormal endoscopy, elevated serum CRP, low serum Hg, and low serum albumin. While it was a small cohort that had imaging, FCAL results were not significantly associated with cross-sectional imaging evidence of disease activity, likely reflecting a weaker association with small bowel disease. Our study confirms that FCAL can be used as a surrogate marker for disease activity in IBD. However, clinicians are placing considerable faith in the test even though its role in small bowel CD requires further research. Finally, optimizing adherence to stool testing in adults is needed. If persons with IBD do not actually have the test done because they will not submit a stool sample, FCAL will not function as a useful predictor of disease activity.

## Figures and Tables

**Table 1:** Patients' characteristics.

Number		183

Diagnosis	UC	72
	CD	107
	Pouchitis	3
	IBD-U	1

Gender	Female	115
	Male	68

Age (years)	Median	24
	Mean	31.3
	Range	4–84

Physician global assessment	Remission	63
	Mild	85
	Moderate	32
	Severe	3

UC phenotype	E1	9
	E2	13
	E3	50

CD phenotype	Location	
	Small bowel	44
	Colon	13
	SB-colon	50
	Upper GI	20
	Perianal	21
	
	Behavior	
	Inflammatory	65
	Stricturing	26
	Fistulizing	16

**Table 2:** Characteristics of adult returners versus nonreturners.

		Adult patients' characteristics that did not do the test		Adult patients' characteristics that did the test	
Return rate	67%	*n* = 49		*n* = 101	

Age	Median	38		47	
	Mean	39.96		46.05	
	Range	18–82		18–84	

Gender	Female	31	63.26%	65	64%
	Male	18	36.73%	36	36%

Diagnosis	CD	28	49.57%	57	56%
	UC	21	40.43	44	44%

PGA	Remission	13		33	
	Mild	29		53	
	Moderate	6		14	
	Severe	1		1	

**Table 3:** Correlation of small bowel—CD with endoscopy and radiography (*n* = 18).

Diagnostic test	Disease activity	FCAL positive	FCAL negative	*P* value
Endoscopy	Active	3	2	0.39
	Inactive	2	4^*∗*^	

MRE/CTE	Active	2	3	0.73
	Inactive	2	3	

^*∗*^3 persons also had video capsule endoscopy.

**Table 4:** Association of FCAL results with serological markers, endoscopy, and radiography.

Diagnostic test	Disease activity	FCAL positive	FCAL negative	*P* value	Odds ratio
					95% CI
MRE/CTE	Active	6	3	0.31	
	Inactive	5	9		

Endoscopy	Active	17	4	0.01	8.79
	Inactive	4	9		1.54–65.55

Albumin	Low	17	3	0.015	4.66
	Normal	71	59		1.26–26.06

Hg	Low^*∗*^	40	16	0.0059	2.74
	Normal	48	53		1.31–5.96

CRP	Elevated	28	8	0.006	3.46
	Normal	48	48		1.36–9.73

PGA	Remission	21	42	0.00004	3.68
	Active	78	42		1.86–7.48

^*∗*^Low hemoglobin (Hg, anemia) is defined as per our local laboratory values which is consistent with WHO definition of anemia with one difference that adult male Hg < 130 g/L (male age ≥ 15 yr Hg < 140 g/L, female age ≥ 15 yr Hg < 120 g/L, children 12–14 yr Hg < 120 g/L, and children 4–11 yr Hg < 115 g/L).

(i) PGA: Physician's global assessment.

**Table 5:** Impact of FCAL results on IBD management.

FCAL test results	Total number	Change in therapy		Change in investigation		Overall impact on management	
		Number	%	Number	%	Number	%
Positive	99	65	65.7	27	27.3	86	86.9
Negative	84	NA	NA	NA	NA	74	88.1
